# Exploring the Interactions Between Neurophysiology and Cognitive and Behavioral Changes Induced by a Non-pharmacological Treatment: A Network Approach

**DOI:** 10.3389/fnagi.2021.696174

**Published:** 2021-07-29

**Authors:** Víctor Rodríguez-González, Carlos Gómez, Hideyuki Hoshi, Yoshihito Shigihara, Roberto Hornero, Jesús Poza

**Affiliations:** ^1^Biomedical Engineering Group, Universidad de Valladolid, Valladolid, Spain; ^2^Centro de Investigación Biomédica en Red en Bioingeniería, Biomateriales y Nanomedicina (CIBER-BBN), Madrid, Spain; ^3^Precision Medicine Centre, Hokuto Hospital, Obihiro, Japan; ^4^IMUVA, Instituto de Investigación en Matemáticas, Universidad de Valladolid, Valladolid, Spain

**Keywords:** non-pharmacological treatment (NPT), Mini-Mental State Examination (MMSE), Dementia Behavior Disturbance Scale (DBD-13), magnetoencephalography (MEG), networks, predict

## Abstract

Dementia due to Alzheimer’s disease (AD) is a neurological syndrome which has an increasing impact on society, provoking behavioral, cognitive, and functional impairments. AD lacks an effective pharmacological intervention; thereby, non-pharmacological treatments (NPTs) play an important role, as they have been proven to ameliorate AD symptoms. Nevertheless, results associated with NPTs are patient-dependent, and new tools are needed to predict their outcome and to improve their effectiveness. In the present study, 19 patients with AD underwent an NPT for 83.1 ± 38.9 days (mean ± standard deviation). The NPT was a personalized intervention with physical, cognitive, and memory stimulation. The magnetoencephalographic activity was recorded at the beginning and at the end of the NPT to evaluate the neurophysiological state of each patient. Additionally, the cognitive (assessed by means of the Mini-Mental State Examination, MMSE) and behavioral (assessed in terms of the Dementia Behavior Disturbance Scale, DBD-13) status were collected before and after the NPT. We analyzed the interactions between cognitive, behavioral, and neurophysiological data by generating diverse association networks, able to intuitively characterize the relationships between variables of a different nature. Our results suggest that the NPT remarkably changed the structure of the association network, reinforcing the interactions between the DBD-13 and the neurophysiological parameters. We also found that the changes in cognition and behavior are related to the changes in spectral-based neurophysiological parameters. Furthermore, our results support the idea that MEG-derived parameters can predict NPT outcome; specifically, a lesser degree of AD neurophysiological alterations (i.e., neural oscillatory slowing, decreased variety of spectral components, and increased neural signal regularity) predicts a better NPT prognosis. This study provides deeper insights into the relationships between neurophysiology and both, cognitive and behavioral status, proving the potential of network-based methodology as a tool to further understand the complex interactions elicited by NPTs.

## Introduction

Dementia is a neurological syndrome that induces cognitive, behavioral, and functional alterations (Cummings, 2003). It is estimated that, in 2019, about 50 million people suffered from dementia worldwide, and this number is expected to increase to 132 million in 2050 (Alzheimer’s Disease International, 2019). Furthermore, its global economic impact is currently estimated at $1 trillion, and it is expected to be doubled by 2030 (Alzheimer’s Disease International, 2019). Alzheimer’s disease (AD) is the most common cause of dementia, with an exponentially growing incidence, especially in developed countries, due to the increase in life expectancy (Alzheimer’s Association, 2019). These figures show that AD is becoming a problem of utmost importance, highlighting the need to develop new treatments to help ameliorate the increasing impact of AD.

Some pharmacological treatments for AD have been developed over the past few years (Alzheimer’s Disease International, 2019). Nonetheless, their effectiveness to mitigate dementia symptoms is very limited and patient-dependent and, in addition, they are often expensive (Qaseem et al., 2008; Alzheimer’s Association, 2019; Alzheimer’s Disease International, 2019). On the other hand, non-pharmacological treatments (NPTs) are showing promising results when dealing with AD-related cognitive alterations (Zucchella et al., 2018; Alzheimer’s Association, 2019). NPTs include a wide variety of strategies, ranging from physical training to cognitive stimulation, through psychological therapy (Dyer et al., 2018). As pharmacological therapies, they are not able to repair or stop the neuronal death caused by AD, but they are beneficial to patients with the disease (Dyer et al., 2018; Alzheimer’s Association, 2019). NPTs have been proven to effectively treat behavioral and psychological dementia symptoms, as well as to improve cognitive function and scores in depression tests (Oliveira et al., 2015; Dyer et al., 2018; Alzheimer’s Association, 2019). Therefore, NPTs are recommended as first-line managers to cope with behavioral and psychological symptoms of dementia, as they do not have adverse effects (Dyer et al., 2018). Nonetheless, their effectiveness has been shown to be patient-dependent (Kurz et al., 2011; Maki et al., 2018; Alzheimer’s Association, 2019). Many factors could influence the outcome of NPTs, such as previous cognitive level, symptom severity, or anti-psychotic use, but their impact is still unclear (Hsu et al., 2017). Therefore, being able to *a-priori* predict NPT outcome is a problem of paramount importance, since it would lead to personalized treatments and, consequently, to increased treatment efficiency.

Neuroimaging techniques could be useful in this regard. They record neuronal activity on different levels, providing a quantitative framework to assess NPT influence on higher cognitive functions. Resting-state electroencephalography (EEG) and magnetoencephalography (MEG) have already been proven to be sensitive to changes induced by NPTs in brain activity (Amjad et al., 2019; Shigihara et al., 2020a,b), as well as to be potential predictors of NPT outcome (Amjad et al., 2019; Shigihara et al., 2020a,b). Both EEG and MEG are noninvasive neurophysiological techniques, though only MEG provides simultaneously high spatial and temporal resolution, as well as low distortion of scalp recordings due to the resistive properties of brain structures (Babiloni et al., 2009). MEG records brain activity in the range of milliseconds, which is of paramount importance to understand the function of a dynamic system like the brain (Babiloni et al., 2009). MEG recordings, and specifically resting-state signals, are often analyzed in patients with AD because they are able to detect the subtle changes that the disease provokes in neural activity (Engels et al., 2017; Mandal et al., 2018). Likewise, as previously mentioned, past studies found individual correlations between MEG-based parameters in specific brain regions and both, cognitive and behavioral variations due to NPTs (Amjad et al., 2019; Shigihara et al., 2020a,b). These results support the potential of MEG to quantify the effects of these therapeutic interventions. In the current research, we propose to further explore the complex interactions between the diverse variables under study by means of a network-related framework, which enables us to glimpse the footprint of the therapy in neural signals in a comprehensive and intuitive way. This approach is based on the generation of the so-called “association networks” that simplify the interpretation of the complex interactions between variables of diverse nature (Borsboom and Cramer, 2013; Fornito et al., 2016; Borsboom, 2017). Association networks are increasingly used as a tool for conceptualizing the interactions between symptoms in mental disorders, given their ability to capture all the intriguing complexity of these pathologies (Borsboom and Cramer, 2013; Borsboom, 2017). To the best of our knowledge, this is the first time that a network framework has been applied to assess the complex associations due to an NPT between neurophysiology, cognition, and behavior in AD. This framework provides a powerful tool to analyze the impact of NPT on neurophysiological signals and its potential predictors in a simple and integrated way.

In this work, we hypothesize that NPT elicits several changes in different cognitive and behavioral dimensions, which in turn modify functional brain activity. The relationships between brain function and higher-order capacities are governed by a complex pattern of interactions between neurophysiological, cognitive, and behavioral variables. Consequently, new methodological approaches are needed to identify the changes in oscillatory brain activity that could be used to quantitatively assess NPT outcomes and, eventually, to design personalized therapeutic interventions. To address these issues, 19 patients with AD went through an NPT. Resting-state MEG activity, cognitive state, and behavioral status were evaluated at the beginning and end of the NPT. Different spectral and non-linear parameters of the MEG recordings were calculated to evaluate their interactions with the NPT outcome, which was measured by means of cognitive and behavioral tests. Specifically, we will address the following research questions: (i) are the association networks able to reflect the influence of the NPT on the relationships between neurophysiological and cognitive/behavioral variables?; (ii) what are the particular changes in the structure of the association networks due to the NPT?; and (iii) can the neurophysiological parameters predict the cognitive and behavioral changes associated with the NPT?

## Materials and Methods

### Participants

Nineteen patients with dementia from the geriatric health services facility “Kakehashi” (Obihiro, Japan) were recruited for this study. It is an official facility authorized by the Ministry of Health, Labor, and Welfare in Japan, recognized as a transient facility between hospitals and patients’ homes. The main role of this facility is to improve the physical and cognitive conditions of aged individuals to enable them to return to their homes. All the participants were diagnosed with AD, and two of them were also diagnosed with other pathologies: one with Parkinson’s disease, and the other one with vascular dementia. The diagnoses were carried out by clinicians before admission in the “Kakehashi” facility, and according to the National Institute on Aging-Alzheimer’s Association criteria (McKhann et al., 2011). If possible, patients’ medication remained unchanged during the NPT period.

**Figure 1:**
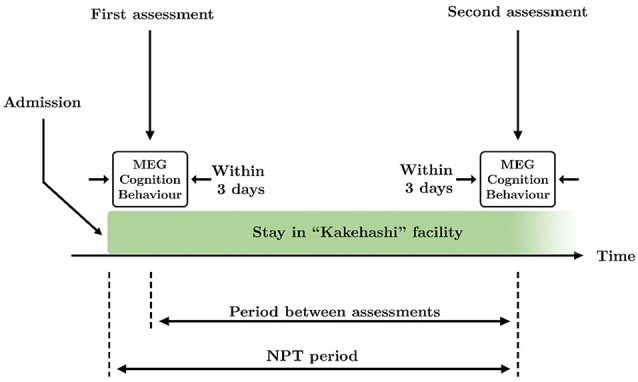
Schematic overview of the time course of the study. Two assessments took place during the study: at the beginning and the end of the therapy. Each assessment consisted of MEG recording and application of Mini-Mental State Examination (MMSE) and Dementia Behavior Disturbance (DBD)-13 tests, that were performed within 3 days. The period between assessments is defined as the time between the first and the second assessments. The non-pharmacological treatment (NPT) period is defined as the time between the admission in the facility and the second assessment.

Patients underwent the NPT for 83.1 ± 38.9 days (mean ± standard deviation, see Figure 1 for a graphical description of the NPT period), being treated every day by the NPT professionals. NPT is composed of five types of activities commonly used in geriatric health services facilities in Japan:

1. **Physical training**. It is aerobic exercise and resistance training, which are effective to improve cognitive function in aged individuals (Nagamatsu et al., 2012; Amjad et al., 2019).
2. **Therapeutic role-playing**. This therapeutic intervention is called “Otona-no-gakko” (“School for adults”) and it is used to both re-introduce patients to active life and enhance their daily motivation (Cotelli et al., 2012).
3. **Nursing care**. Nursing care provides proper eating, drinking, and a sanitary environment, which are essential to keep brain activity healthy. Furthermore, it has been previously suggested that diet has some relevant impact on AD (Rege et al., 2016; McGrattan et al., 2019).
4. **Horticultural therapy**. This therapy is based on gardening and planting activities to improve physical and cognitive conditions (Lu et al., 2020). Patients were familiar with these activities since our facility is located in an agricultural area.
5. **Self-cognitive training**. Self-cognitive training includes activities such as coloring books or crossword puzzles (Anderson and Grossberg, 2014).

Each NPT session was designed each day by the experts to adapt it to the clinical features and mood of each patient (Maki et al., 2018), with a duration ranging from 20 to 40 min, according to the Japanese regulations. See Table 1 for a description of the sociodemographic and clinical information of the sample.

**Table 1:** Sociodemographic and clinical information of the sample.

Sociodemographic data		
**Number of subjects**	19
**Age (years)**	86.00 ± 3.86
**Gender (M:F)**	7:12
**NPT Period (days)**	83.05 ± 38.88
	**Pre**	**Post**
**MMSE**	14.11 ± 5.95	16.00 ± 7.32
**DBD-13**	10.89 ± 9.93	9.84 ± 10.55

*Data are shown as mean ± standard deviation. M, male; F, female; MMSE, Mini-Mental State Examination; DBD-13, Dementia Behavior Disturbance scale*.

All participants and their families or caregivers gave their informed consent to participate in the present study. The investigation was carried out in accordance with the Code of Ethics of the World Medical Association (Declaration of Helsinki). The protocol was approved by the Ethics Committee of Hokuto Hospital.

### Cognitive and Behavioral Assessment

Cognitive and behavioral performance was assessed twice for each patient, at the beginning and at the end of the NPT. Each assessment session consisted of two different tests conducted on the same day: an abbreviated version of the Dementia Behavior Disturbance Scale (DBD-13; Machida, 2012), and the Japanese Mini-Mental State Examination (MMSE; Folstein et al., 1975; Sugishita et al., 2010). The DBD-13 scale is a 52-point test consisting of 13 items of the original DBD-28 scale (Baumgarten et al., 1990; Machida, 2012). It measures the behavioral disturbance induced by dementia, assigning higher values to more behavior problems. The MMSE is a test with a maximum score of 30, which measures cognitive impairment by assessing different cognitive domains (Folstein et al., 1975; Sugishita et al., 2010). Lower values correspond to more impaired cognition.

### MEG Recordings

All MEG recordings were acquired at the Hokuto Hospital (Obihiro, Japan). As for the cognitive and behavioral assessments, brain signals were recorded twice: at the beginning and end of the treatment. MEG recordings, cognitive state, and behavioral status were evaluated within 3 days in order to: (i) accurately match each MEG recording with a cognitive and behavioral assessment; and (ii) make the intervals between MEG recordings and both cognitive and behavioral assessments as similar as possible. Thereby, 76.1 ± 36.0 days (mean ± standard deviation) passed between the initial and final assessments. See Figure 1 for a graphical description of the period between assessments.

For each subject, 5 min of resting-state brain activity was recorded using a 160-channel axial gradiometers MEG system (MEG Vision PQ1160C, Yokogawa Electric), with a sampling rate of 1,000 Hz and a low-pass filter at 200 Hz. Head position was registered with three fiducial markers placed on the patient’s head during the MEG scan: 5 mm above the nasion, and 10 mm in front of the tragus on each side of the head. Patients were asked to stay calm and awake with eyes closed, in a supine position during the recording. For security reasons, as well as to prevent somnolence, MEG recordings were monitored in real time.

### MEG Analysis

Signals were preprocessed before the application of the source inversion algorithm. Next, different spectral and non-linear local activation parameters were calculated from the signals at the source level. Finally, these parameters were used to construct the networks based on the Spearman correlations between them. The next subsections describe the steps followed in the MEG analysis in detail.

#### Preprocessing of MEG Signals

To limit the presence of noise in the MEG recordings, signals were preprocessed using a 4-step pipeline (Rodríguez-González et al., 2020): (i) artifact removal using the SOUND algorithm (Mutanen et al., 2018; Rodríguez-González et al., 2019); (ii) finite impulse response (FIR) filtering: 1–70 Hz band-pass to limit noise bandwidth, and 49–51 Hz band-stop to remove line noise; (iii) artifact removal using independent component analysis; and (iv) visual selection of 5-s artifact-free epochs.

#### Source Inversion

Source-level signals were obtained using the Brainstorm toolbox, which is documented and freely available for download online under the GNU general public license^1^ (Tadel et al., 2011). A forward model with 15,000 sources was created by means of boundary element model using the ICBM152 head template (Montreal Neurological Institute) and OpenMEEG software (Fonov et al., 2009; Gramfort et al., 2010; Douw et al., 2018). The head model was segmented into three tissues: brain, skull, and scalp, with conductivities of 1, 0.0125, and 1 Siemens per meter, respectively (Mahjoory et al., 2017). Sources were restricted to the cortex, and their direction was set normal to it (Mahjoory et al., 2017; Lai et al., 2018; Rodríguez-González et al., 2020). No noise recordings were available, so an identity matrix was used as noise covariance (Lai et al., 2018; Rodríguez-González et al., 2020). The 15,000 source-level time courses were projected into the 68 regions of interest (ROIs) provided by the Desikan-Killiany atlas, in order to have a manageable number of ROIs to work with Desikan et al. (2006), Lai et al. (2018), and Rodríguez-González et al. (2020). This source projection was done by averaging the reconstructed activation time courses of all the voxels in each ROI after flipping the sources of opposite direction (Lai et al., 2018; Rodríguez-González et al., 2020).

As, we were working with resting-state signals, no *a-priori* assumptions about sources could be made. Thus, we used the weighted minimum-norm estimation (wMNE) algorithm, which restricts the solutions by minimizing the energy (*L*_2_ norm) weighting deep sources to facilitate their identification (Lin et al., 2004). This method has been proven to be useful to reconstruct the underlying sources of resting-state MEG datasets (Lin et al., 2004).

#### Feature Extraction

Diverse signal processing methods have been widely used to describe the properties of brain activity. These methods characterize the electromagnetic fields generated by the synchronized neuronal pools responsible for the observed brain activity. In this study, we have used several local activation parameters, which measure the activation of single functional units (i.e., synchronized neuronal pools; Stam and van Straaten, 2012) They can be grouped in two main categories: (i) spectral parameters, which evaluate the time-frequency content of the recorded signal; and (ii) non-linear parameters, which measure relevant non-linear properties of the signal, such as variability, irregularity, or complexity. In this study, we have calculated a wide variety of parameters in both categories to fully characterize the properties of the resting-state MEG activity using its source reconstructed time courses on the 68 estimated ROIs.

##### Spectral Parameters

They are useful to characterize the spectral content of the signal. They were derived from the normalized power spectral density (PSDn), which was calculated using the Blackman-Tuckey method (Blackman and Tukey, 1958; Ruiz-Gómez et al., 2018; Rodríguez-González et al., 2020). The parameters computed in this category are listed below:

- **Relative power (RP)**. It summarizes the neural activation in a certain frequency range, relative to the full spectral content of the signal. RP was calculated in the well-known conventional frequency bands: delta (δ, 1–4 Hz), theta (θ, 4–8 Hz), alpha (α, 8–13 Hz), beta 1 (β_1_, 13–19 Hz), beta 2 (β_2_, 19–30 Hz), and gamma (γ, 30–70 Hz).
- **Median frequency (MF)**. MF is the frequency that divides the PSDn into two halves of equal power. It is commonly used to measure the global signal slowing provoked by AD disruptions (Poza et al., 2007; Dauwels et al., 2011).
- **Individual alpha frequency (IAF)**. It measures the frequency where the alpha peak can be found. It is calculated as the frequency that divides the extended alpha band (4–15 Hz) into two halves of equal power (Klimesch, 1999; Poza et al., 2007). Alpha peak is related to higher cognitive functions, so this parameter is widely used to assess cognitive disfunction (Klimesch, 1999; Poza et al., 2007).
- **Spectral entropy (SE)**. This parameter measures the flatness or uniformity of the PSDn using Shannon entropy. It has been proven that patients with AD show a less distributed spectral content of the PSDn than controls, which suggests less variety of neural oscillatory components (Poza et al., 2008b; Gómez and Hornero, 2010).
- **Spectral edge frequency (SEF)**. It is quantified as the upper limit of the PSDn. This parameter is calculated as the frequency that comprises 95% of the power of the PSDn, and is identified as the bandwidth of the signal (Poza et al., 2007). Due to the slowing and the reduction in the variety of neural oscillatory activity associated with AD, this parameter has been used to characterize brain signals in patients with dementia (Poza et al., 2007).

##### Non-linear Parameters

Non-linearity is a fundamental property of complex systems, such as the brain (Stam, 2005). Non-linear analyses of brain signals are then commonly used to describe the alterations produced by a neuropathology like AD. The non-linear parameters assessed in this study are:

- **Lempel-Ziv complexity (LZC)**. It is a coarse-grain complexity measure. LZC estimates the complexity by counting the number of subsequences that the binarized version of the analyzed signal contains (Lempel and Ziv, 1976). It assigns higher values to more complex time series (Fernández et al., 2010, 2011). A decrease in complexity has been associated with AD progression (Gómez et al., 2006; Fernández et al., 2010).
- **Sample entropy (SampEn)**. SampEn is an irregularity measure that assigns higher values to more irregular time sequences. It has two tuning parameters: the sequence length and the tolerance, which were respectively set to 1 and 0.25·std (std: standard deviation of the signal) based on previous studies (Gómez et al., 2009; Hornero et al., 2009; Rodríguez-González et al., 2020). A decrease in irregularity has been observed in the neural activity of patients with AD (Escudero et al., 2009; Gómez et al., 2009; Hornero et al., 2009).
- **Central tendency measure (CTM)**. This parameter is useful to quantify the variability of a signal. It is based on calculating the second-order differences diagram of the time series and then counting the points within a radius. In the present study, the radius has been set to 0.025, based on previous analyses (Ruiz-Gómez et al., 2018; Rodríguez-González et al., 2020). CTM assigns higher values to less variable signals. Previous studies have reported that AD is associated with lower CTM values (Ruiz-Gómez et al., 2018).

In addition to the spectral and non-linear parameters, a new measure is presented in the current study: the spatial Shannon entropy (SSE). Specifically, the SSE computes the entropy of the spatial distribution of values for a given local activation parameter. The spatial distribution of the considered parameter is estimated as the normalized histogram of its values considering the 68 ROIs. The calculation of the SSE of a local activation parameter enables us to quantify the changes induced by the NPT in the spatial patterns of brain oscillatory activity. A parameter with similar values across the brain (i.e., showing a delta-like distribution of values) would yield a low SSE value, whereas a high SSE value would be obtained by a parameter with a wide range of variation (i.e., displaying a uniform distribution). It is noteworthy that in the previous examples the parameters could have similar mean values, but their SSE values would be different. Hence, the SSE was computed for each spectral and non-linear parameter; the SSE of a given parameter will be referred to as S with the parameter name in brackets, e.g., the SSE of the IAF will be denoted as S(IAF).

#### Construction of Association Networks

In this study, we have generated different networks to account for the potential relationships between the neurophysiological, cognitive, and behavioral parameters. Thereby, the network nodes were individual variables (all the neurophysiological parameters, the score in the cognitive examination—i.e., MMSE–, and the score in the behavioral test—i.e., DBD-13), and the network edges (or weights) were the associations between them. These associations were estimated as the Spearman rank correlations between pairs of variables to detect both linear and non-linear monotonic interactions; age and gender were introduced in the correlation analysis as covariates to control for their effect. Non-significant correlations (i.e., network edges with *p*-values > 0.05) were removed from the network (Zhang, 2011; Barberán et al., 2012). For the sake of simplicity, negative correlations were converted to positive, as we were interested in the association, and not in the nature of that association. Afterward, networks were constructed using Gephi software^2^. The width of the edges was linked to the magnitude of the relationship, with a wider edge meaning stronger association. The Force Atlas 2 algorithm was employed to group nodes with higher correlations while taking nodes with lower correlations away (Jacomy et al., 2014). No regularization algorithm was applied, as we were interested in exploring all the associations, especially those of cognitive and behavioral parameters, even if they are weaker than others.

Four different associations networks were generated:

- **Pre-NPT network**. The nodes were the neurophysiological, cognitive, and behavioral parameters obtained before the application of the NPT.
- **Post-NPT network**. The nodes were the neurophysiological, cognitive, and behavioral parameters obtained after the application of the NPT.
- **Changes network**. The nodes were the variation of neurophysiological, cognitive, and behavioral parameters during the NPT, i.e., the difference between their values before and after the NPT.
- **Prediction network**. The nodes were the neurophysiological parameters before the application of the NPT, and the variation of the cognitive and behavioral parameters during the NPT.

Furthermore, networks stability was analyzed. To do that, the probability of obtaining similar results was assessed by using a bootstrapping methodology with 2,000 iterations to generate new random network instances (Efron, 1992; Epskamp et al., 2018). In each iteration, a new network is created by randomly selecting 19 subjects of the original dataset, being possible to select the same subject more than once (Efron, 1992). From the four bootstrapped instances (one for each network), the 95% confidence interval of the edges’ weights was reported (Jimeno et al., 2020).

Another network was generated using the bootstrapped samples from Pre-NPT and Post-NPT networks: the **Variability network**. This network shows the differences in the association pattern between Pre-NPT and Post-NPT. Each network edge from the Variability network was computed as the test statistic obtained when comparing the bootstrapped samples from Pre-NPT and Post-NPT networks for that particular edge. Thus, the higher the edge weight in the Variability network, the higher the differences between Pre-NPT and Post-NPT networks. Although all the edge weights were statistically significant (*p*-values < 0.05, Wilcoxon signed rank test), only the 5% strongest connections (i.e., showing the biggest differences between Pre-NPT and Post-NPT networks) were displayed.

## Results

### Changes in Cognition and Behavior Induced by the NPT

The average MMSE value before conducting the NPT was 14.11 ± 5.95 (mean ± SD), while the average for DBD-13 was 10.89 ± 9.93. Then, after applying the NPT, the average value for the MMSE was 16.00 ± 7.32, and the average for DBD-13 9.84 ± 10.55. The effectiveness of the NPT was assessed by comparing the MMSE and DBD-13 before and after conducting the NPT. Both, MMSE and DBD-13 show a statistically significant improvement after the NPT, with MMSE significantly increasing (*p*-value = 0.0323, one-tailed Wilcoxon signed rank test) and DBD-13 significantly decreasing (*p*-value = 0.007, one-tailed Wilcoxon signed rank test).

### Changes in Network Structure Induced by the NPT

**Figure 2:**
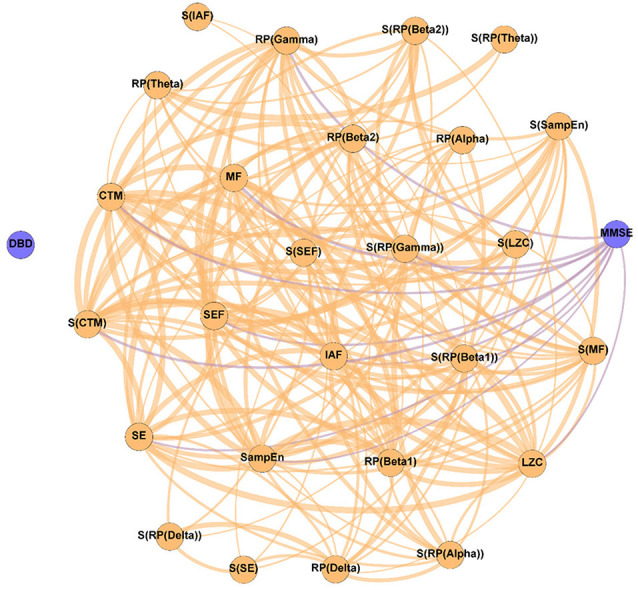
Parameter network of the patients with Alzheimer’s disease (AD) before the NPT (Pre-NPT network). Wider edges correspond with stronger associations. Nodes corresponding to cognitive and behavioral parameters are shown in blue, while nodes of neurophysiological parameters are shown in orange.

**Figure 3:**
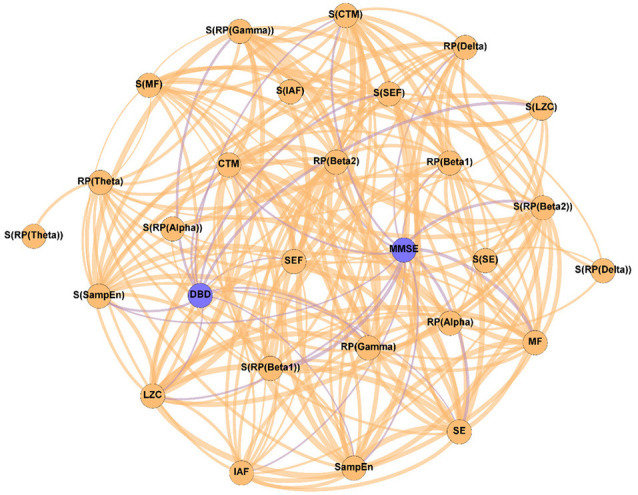
Parameter network of the patients with AD after the NPT (Post-NPT network). Wider edges correspond with stronger associations. Nodes corresponding to cognitive and behavioral parameters are shown in blue, while nodes of neurophysiological parameters are shown in orange.

**Figure 4:**
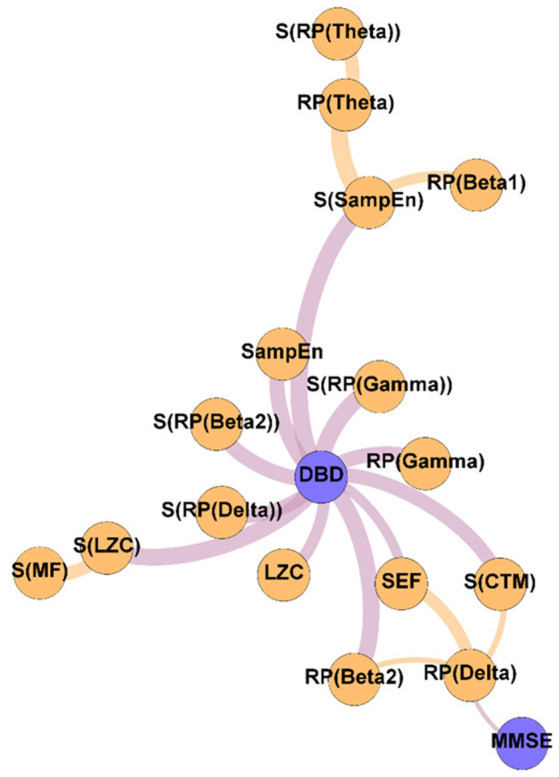
Variability network, showing the 5% strongest connections corresponding to the network edges that obtained the most significant differences between Pre-NPT and Post-NPT networks. Wider edges correspond with stronger associations. Nodes corresponding to cognitive and behavioral parameters are shown in blue, while nodes of neurophysiological parameters are shown in orange.

In order to evaluate the changes induced by the NPT in the patients’ network structure, three different networks were evaluated: (i) Pre-NPT network (Figure 2); (ii) Post-NPT network (Figure 3); and (iii) Variability network (Figure 4). The figures depicting the stability of the networks shown in Figures 2, 3 can be found in the Supplementary Figures 1, 2.

Figure 2 displays the relationships between the parameters under study without the influence of the NPT, as they were calculated with the samples obtained at the beginning of the therapeutic intervention. It can be observed that while DBD-13 is disconnected (i.e., it has no relationship with any other parameter), MMSE is related with another 11 parameters: RP(Gamma), MF, IAF, SE, SEF, LZC, SampEn, CTM, S(RP(Gamma)), S(RP(Beta 1)), and S(CTM). Interestingly, no associations were observed for any RP parameter apart from RP(Gamma).

On the other hand, Figure 3 shows the association network, but including the influence of the NPT, as it has been calculated with the parameters obtained after conducting the NPT. The Post-NPT network has a higher number of connections on cognitive and behavioral parameters in comparison with the Pre-NPT network: MMSE now has 13 connections, while DBD-13 has 11. Of note, eight out of the 13 associations of MMSE were maintained from the Pre-NPT network (MF, IAF, SE, LZC, SampEn, CTM, S(RP(Beta 1)), and S(CTM)), while the other five were new associations (RP(Delta), RP(Beta 1), RP(Beta 2), S(RP(Beta 2)), and S(SampEn)). In contrast to the Pre-NPT network, three parameters based on RP are now associated with the MMSE, but RP(Gamma) is no longer significant. Furthermore, the significant relationships for DBD-13 that can be appreciated in the Post-NPT network are with: RP(Gamma), SE, SEF, LZC, SampEn, CTM, S(RP(Gamma)), S(SEF), S(LZC), S(SampEn), and S(CTM).

To get deeper insights on the changes induced by the NPT in the parameter network, the Variability network was constructed, depicting the 5% strongest differences between Pre-NPT and Post-NPT networks (Figure 4). As expected, the parameter whose relationships have changed most between both networks is DBD-13, with 11 connections, while MMSE only showed one connection. Interestingly, six out of those 11 connections (S(RP(Delta), S(RP(Beta 2)), S(RP(Gamma)), S(LZC), S(SampEn), and S(CTM)) are spatial entropies.

### Relationship Between Neurophysiological and Cognitive and Behavioral Changes

**Figure 5:**
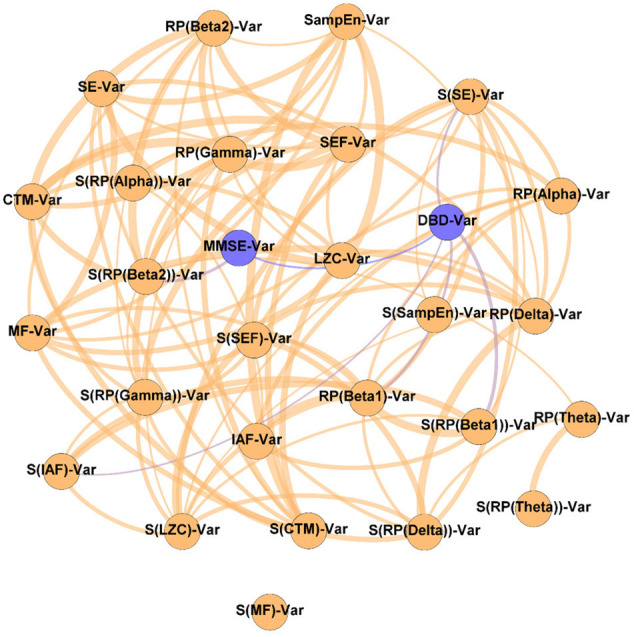
Network of changes. As indicated by the “Var” suffix, this network displays the relationships between the variation of the parameters under study during the NPT (i.e., Parameter_Post_ − Parameter_Pre_). Wider edges correspond with stronger associations. Nodes corresponding to cognitive and behavioral parameters are shown in blue, while nodes of neurophysiological parameters are shown in orange.

The network of changes can be observed in Figure 5. This network describes the associations between the variation of the parameters under study (neurophysiological, cognitive, and behavioral) by computing: Parameter_Post_ − Parameter_Pre_. A positive value indicates an increase in the parameter provoked by the NPT, while a negative value is associated with a decrease. The figure showing the stability of the network depicted in Figure 5 can be found in the Supplementary Figure 3.

It could be observed that apart from the DBD-13 - MMSE relationship, DBD-13 displays four connections RP(Beta 1), S(IAF), S(SE), and S(RP(Beta 1)), while MMSE only one S(RP(Beta 2)). Interestingly, four out of five significant associations involve spatial entropies: S(RP(Beta 1)), S(IAF), S(SE) with DBD, and S(RP(Beta 2)) with MMSE. Besides, three associations involve beta band: RP(Beta 1), S(RP(Beta 1)), and S(RP(Beta 2)). No association involves any non-linear parameter.

### Predictability of the NPT Outcome by Means of the Neurophysiological Parameters

**Figure 6:**
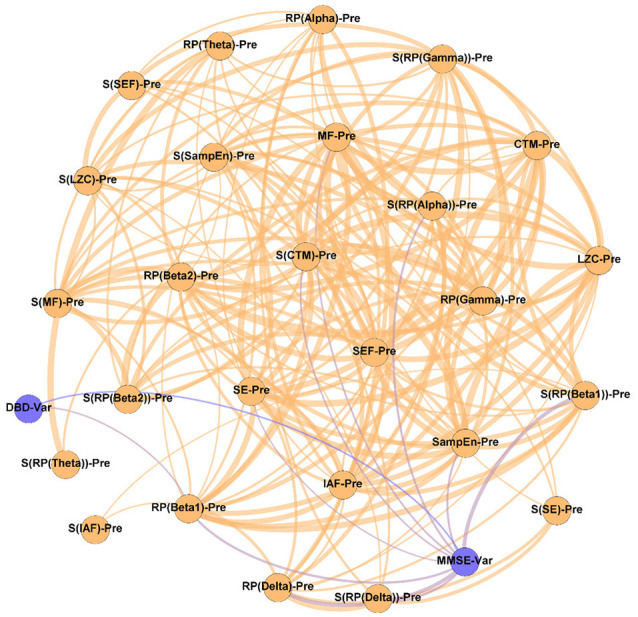
Prediction network, showing the ability of the assessed neurophysiological parameters to predict the outcome of the NPT. The “Var” suffix, which appears associated with DBD and MMSE, means the variation of the parameters under study during the NPT (i.e., Parameter_Post_ − Parameter_Pre_). Wider edges correspond with stronger associations. Nodes corresponding to cognitive and behavioral parameters are shown in blue, while nodes of neurophysiological parameters are shown in orange.

Figure 6 contains the Prediction network. It depicts the ability of the neurophysiological parameters under study to predict the outcome of the NPT, measured by the variation of the cognitive and behavioral parameters (MMSE and DBD-13). It could be appreciated that, aside from the relationship that links cognitive and behavioral parameters, MMSE has nine significant relationships (RP(Delta), RP(Beta 1), MF, SE, SampEn, S[RP(Delta)], S[RP(Alpha)], S(RP(Beta 1)), and S(CTM)), while DBD-13 has only one (RP(Beta 1)). Of note, only two out of these 10 parameters involve non-linear parameters (MMSE-SampEn and MMSE-S(CTM)), and four of them involve spatial entropies (S[RP(Delta)], S[RP(Alpha)], S(RP(Beta 1)), and S(CTM)). Supplementary Figure 4 depicts the stability of the network depicted in Figure 6.

**Figure 7:**
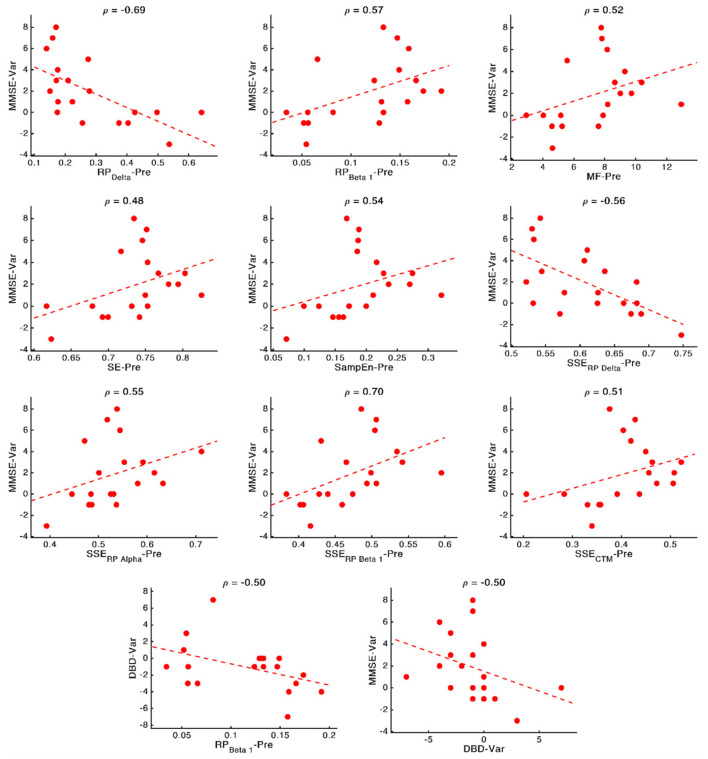
Scatterplots representing the relationship between the neurophysiological parameters computed before applying the NPT (x-axis) and the changes in cognitive and behavioral variables after the NPT (y-axis). In the top part of each panel, the value of the Spearman rank correlation for each specific pair of parameters is plotted. Dashed lines represent the linear regression of the data.

These relationships are of great importance because, as mentioned in the Introduction section, predicting the NPT is crucial. Thus, to obtain deep insights on them, and disentangle the nature of these associations, we plotted scatterplots for every significant relationship (involving cognitive or behavioral parameters) obtained in the previous section, reporting the specific correlation values (ρ). These scatterplots are shown in Figure 7. Remarkably high relationships between parameters can be observed, with a mean value of 0.56. The strongest relationships can be observed for associations involving MMSE and delta and beta bands: MMSE-RP(Delta) (*ρ* = −0.69, *p*-value = 0.002, Spearman rank correlation), MMSE-RP(Beta 1) (*ρ* = 0.57, *p*-value = 0.017, Spearman rank correlation), MMSE-S(RP(Delta)) (*ρ* = −0.56, *p*-value = 0.019, Spearman rank correlation), and MMSE-S(RP(Beta 1)) (*ρ* = 0.70, *p*-value = 0.002, Spearman rank correlation).

## Discussion

In the present study, we have assessed the effects of an NPT in the neurophysiology of patients with AD, as well as whether its outcome is predictable by means of MEG-based parameters. Our results hold three main findings related to the three research questions posed in the introduction: (i) the NPT alters the structure of the association networks, unveiling relationships between DBD-13 and neurophysiological parameters: RP(Gamma), SE, SEF, LZC, SampEn, CTM, S(RP(Gamma)), S(SEF), S(LZC), S(SampEn), and S(CTM); (ii) the changes induced by the NPT are related to the changes in the DBD-13, suggesting an impact of the NPT in the behavioral symptoms of AD; and (iii) the value of nine neurophysiological parameters (RP(Delta), RP(Beta 1), MF, SE, SampEn, S[RP(Delta)], S[RP(Alpha)], S(RP(Beta 1)), and S(CTM)) before going through the NPT are related to the NPT outcome, suggesting a potential predictive power of the aforementioned parameters to foresee the response of the patients to the NPT.

### NPT Induces Several Changes in the Structure of the Association Networks

The Pre-NPT network displayed in Figure 2 shows that, before conducting the NPT, the neurophysiological parameters are associated with the MMSE, but not with the DBD-13. This could be explained as both tests are measuring the alterations provoked by dementia in different cognitive domains. On the one hand, DBD-13 measures strictly behavioral disturbances defined as “*the outward manifestation of some underlying cognitive, psychological, or physiological deficit—regardless of etiology—likely to cause stress to those caring for the patient*” (Baumgarten et al., 1990). On the other hand, MMSE quantifies cognitive impairment in a more global sense, by means of different cognitive dimensions, such as attention, orientation, language, perception, calculus, or the ability to follow simple instructions (Folstein et al., 1975). Therefore, these results suggest that cognitive disturbances measured in a broader sense are directly related to the neurophysiological state. Nevertheless, this is not the case for behavioral disturbances, where this relationship could be mediated or obscured by external factors, such as the environment, relationship with caregivers, or daily life habits.

It is worth mentioning that all the spectral and non-linear parameters, apart from those derived from the RP (except RP(Gamma)), show statistically significant associations with the MMSE. RP values do not contain information about the complete oscillatory activity, but only of a certain frequency band, typically associated with a limited number of cognitive functions (Uhlhaas et al., 2008). As it is known that AD induces alterations in several cognitive domains (Alzheimer’s Association, 2019; Alzheimer’s Disease International, 2019), it could be hypothesized that the absence of associations between MMSE and RP could be provoked, at least partially, because they are only reflecting particular cognitive dimensions of AD disruptions. Noteworthy, associations were found for RP(Gamma), as well as for its spatial entropy. The gamma band has been proven to play an important role in several higher cognitive functions (Bartos et al., 2007; Martorell et al., 2019). Besides, this frequency band is also affected by AD neuropathology. Previous studies reported that AD patients’ brain activity is associated with an enhanced gamma power (van Deursen et al., 2008; Wang et al., 2017), an increase in long distance gamma connectivity (Başar et al., 2017), and an increase in the cross-frequency-coupling strength between gamma and low frequency bands (Wang et al., 2017).

The Post-NPT network included in Figure 3 depicts the association network after the application of the NPT. By comparing this network with that in Figure 2, we can infer the influence of the NPT in the pattern of interactions between the different parameters (neurophysiological, cognitive, and behavioral) under assessment. In this regard, the Variability network (Figure 4) is also relevant, as it shows the 5% strongest associations with the biggest differences induced by the NPT. For MMSE, the basic structure of the network is relatively maintained, as 62% of the statistically significant associations are the same before and after conducting the NPT. Of note, the associations with RP(Gamma), as well as its spatial entropy S(RP(Gamma)), are not statistically significant; this result can be interpreted as the NPT modulating the impairment provoked by AD in the gamma band. Gamma activity is associated with gamma-aminobutyric acid (GABAergic) activity, which is the principal inhibitory neurotransmitter (Bartos et al., 2007; Porges et al., 2017). Since an increased concentration of GABA is related to superior cognitive performance, we may suggest a relationship between gamma activity and cognitive performance (Bartos et al., 2007; Porges et al., 2017; Mably and Colgin, 2018). Besides, an increase in the gamma band activity of the angular gyrus for AD patients has also been reported (Shigihara et al., 2020b). This increase was associated with the NPT inducing compensatory mechanisms against the functional deficit provoked by dementia (Shigihara et al., 2020b). Furthermore, new associations between MMSE and RP appear with the application of the NPT: RP(Delta), RP(Beta 1), RP(Beta 2), and S(RP(Beta 2)). These bands are associated with the well-documented slowing that AD elicits on oscillatory neural activity (Jeong, 2004; Dauwels et al., 2011); therefore, they are likely to be affected by the NPT. Interestingly, the association of MMSE with RP(Alpha) is missing in both networks, though it is commonly related to AD. This could be due to the fact that the alpha band is acting as a “transition” band between the decrease of power in faster bands (beta 1 and beta 2) and the increase in the slower ones (delta and theta), thus being less affected by the NPT. This result does not agree with previous findings (Shigihara et al., 2020b), where the NPT induced differences in the right temporal and right fusiform areas in the alpha band. The discrepancies could be due to the band definition (alpha band was split in alpha 1 and alpha 2) or due to the spatial dimension of the analyses conducted by Shigihara et al. (2020b).

Crucially, in Figure 4, it could be observed that the majority of the associations that changed the most after the NPT involve the DBD-13. This could be explained because of the environment and habits of the patients being controlled during the NPT, i.e., the therapeutic intervention would be modulating those external factors, that could, in turn, be mediating or obscuring the associations between DBD-13 and the neurophysiological parameters before the application of the NPT. Thus, we can speculate that the NPT has a direct impact on the behavioral disturbances associated with AD, unveiling their association with the neurophysiological oscillatory activity. The behavioral symptoms are common in dementia, and largely affect health and quality of life (Dyer et al., 2018). This is in line with previous studies, where NPTs showed greater effectiveness against behavioral symptoms than against cognitive symptoms (Zucchella et al., 2018). Interestingly, 55% of the associations of DBD-13 that changed the most after the NPT are spatial entropies. This suggests that the spatial patterns of the neurophysiological alterations elicited by NPT play a significant role in patients with AD. Diverse brain regions are affected differently by the NPT and, consequently, the spatial entropy of local activation parameters is able to reflect such changes. This is supported by previous studies where NPT effects were observed in specific brain regions such as the fusiform gyrus, right angular gyrus, sensorimotor area, or right temporal lobe (Zucchella et al., 2018; Shigihara et al., 2020a,b).

### Relationship Between Neurophysiological, Cognitive, and Behavioral Changes

Our findings suggest that changes in RP(Beta 1) and RP(Beta 2) are related with the cognitive and behavioral changes: changes in RP(Beta 1) and S(RP(Beta 1)) are associated with changes in DBD-13, whereas changes in S(RP(Beta 2)) are related with those in MMSE. Beta activity is known to be associated with GABA transmission, somatosensory functions, and emotional processes (Jensen et al., 2005; Poil et al., 2013). Besides, beta oscillations have been linked to AD: a decrease in beta activity associated with the disease has been widely reported (Jeong, 2004; Fernández et al., 2006; Poza et al., 2007; Dauwels et al., 2011; Roh et al., 2011). Hence, its application as a clinical tool to aid in AD diagnosis and to assess neural disruption processes has been proposed (Poil et al., 2013). Likewise, it has also been linked to neuroplasticity, as well as to behavioral and psychological symptoms of dementia *via* GABAergic activity (Lanctôt et al., 2004; Griffen and Maffei, 2014). Also, in previous studies associations between RP in beta and the changes in cognition induced by an NPT has been reported (Shigihara et al., 2020a,b).

Furthermore, a remarkable number of relationships between the spatial entropies and both, cognitive and behavioral parameters can be observed. This fact reinforces the idea posed in the previous section: the NPT not only affects the global values of the parameters under study, but their spatial distribution (i.e., the changes induced by the NPT follow a specific spatial pattern). This could be explained by the NPT restoring specific cognitive domains (Zucchella et al., 2018), which are placed in specific brain regions (Augustine, 2007), and can be detected by cognitive and behavioral tests (e.g., behavior for DBD-13 or memory for MMSE; Folstein et al., 1975; Baumgarten et al., 1990). The NPT affecting different brain regions differently has been previously reported (Zucchella et al., 2018; Shigihara et al., 2020a,b).

Finally, it can also be observed that the changes in non-linear parameters are not related to the changes in cognition or behavior, indicating that the NPT does not directly affect the non-linear properties of resting-state MEG activity. It should be noted that the non-linear parameters are affected by the NPT (as discussed in the previous section), but those changes are not related to the NPT outcome (as measured by the cognitive and behavioral tests). Thus, it is possible that non-linear parameters are affecting specific cognitive domains not measured by the tests, or that those domains are related to specific aspects of the tests, thus blurring those differences among the other dimensions. This is in line with previous studies showing that, although spectral and non-linear parameters are related (Dauwels et al., 2011), they also had remarkable differences. Furthermore, the absence of connections with non-linear parameters could be due to a decreased sensitivity of the non-linear parameters to detect the AD neurophysiological disruptions. This issue is in line with previous studies, where non-linear parameters showed reduced capabilities for AD classification compared to the spectral ones (Hornero et al., 2008; Escudero et al., 2009; Poza et al., 2012).

### Potential of Neurophysiological Parameters to Predict the NPT Outcome

As stated in the Introduction section, predicting the outcome of the NPTs would be of great interest, as important differences have been found in the cognitive impact of NPT among patients; some of them showed great responsiveness to the treatment, while others were unresponsive (Shigihara et al., 2020a).

It can be observed in Figure 6 that spectral parameters are more associated with the NPT outcome than non-linear ones: seven out of the nine local activation parameters that show statistically significant relationships are derived from the PSDn. This suggests that the NPT has a greater influence on the spectral components of the resting-state MEG activity than on its non-linear properties, which could be motivated by greater disruptions of AD in the spectral content than in the non-linear properties of the neural signals (Hornero et al., 2008; Escudero et al., 2009; Poza et al., 2012).

Associations between MMSE and RP(Delta), RP(Beta 1), and their SSEs can be appreciated, though the strongest association was obtained between DBD-13 and RP(Beta 1). These two bands are related to AD, as they measure the well-known frequency shift provoked by AD: an increase of oscillatory activity in low frequency bands and a decrease in higher ones (Jeong, 2004). Not only the beta band, as previously stated, but also the delta band is found to be associated with AD pathology. Delta has been associated with the cholinergic levels of the brain, with the current cognitive status, and also with the progression of AD. Additionally, its increased delta activity has been proposed as evidence of neural degeneration (Fernández et al., 2013; Nakamura et al., 2018; Shigihara et al., 2020a). Furthermore, the ratio between the power of neural activity in delta and beta bands has been used to reflect AD disruptions (Babiloni et al., 2004; Poza et al., 2008a; Knyazeva et al., 2010; Wang et al., 2017). In a previous study, a correlation between beta power and NPT outcome, measured by means of the MMSE, was also observed (Shigihara et al., 2020a).

It is also noteworthy that our results suggest that milder decline (measured by means of the neurophysiological deterioration, i.e., slowing, diminished variety of frequency components and irregularity loss; Jeong, 2004; Escudero et al., 2009; Dauwels et al., 2011) is related with a better NPT outcome. While AD provokes a shift to lower frequencies and a reduced SE (Poza et al., 2008b; Dauwels et al., 2011; Bruña et al., 2012), we have found that a PSDn skewed towards higher frequencies (observed in the correlations MMSE-RP(Delta), MMSE-RP(Beta 1), MMSE-MF, and DBD-13-RP(Beta 1)) and with a richer variety of frequency components (observed in the correlation MMSE-SE) predicts a better outcome of the therapy. Besides, AD is linked with more regular signals (Escudero et al., 2009; Gómez et al., 2009; Hornero et al., 2009), and we have observed that signals with higher irregularity (observed in the correlation MMSE—SampEn) predict a better response of the patient to the therapy. The correlation between DBD-13 and RP(Beta 1) is negative, whereby higher beta power is related to lower DBD-13, which indicates a better behavioral state.

Besides, the SSE of the parameters were shown to be important for predicting the NPT outcome: we found four statistically significant correlations between MMSE and S[RP(Delta)], S(RP(Beta 1)), S[RP(Alpha)], and S(CTM). Also, all the correlations apart from the one with S[RP(Delta)] are positive, which suggests that a more homogeneous spatial distribution of the corresponding local activation parameters predicts a better prognosis for the NPT. AD does not affect the whole brain simultaneously, it is a progressive process (Raji et al., 2009). Thus, a lower SSE could indicate that neural damage is focused on specific brain areas (due to the variations in the spatial pattern of the neurophysiological parameters), which the NPT is unable to recover, so yielding a worse outcome of the therapeutic intervention. Again, this idea is in line with previous findings, where the spatial dimension of the results related to the NPT is evident (Shigihara et al., 2020a,b).

The NPT significatively improved both cognition, as indicated by the MMSE, and behavior, as quantified by the DBD-13. These findings agree with previous studies where other NPTs yielded beneficial effects in dementia patients (Zucchella et al., 2018). Besides, in a previous study with the same NPT but a different sample, statistically significant improvements were observed for the MMSE but not for the DBD-13 (Shigihara et al., 2020b). The discrepancy in the DBD-13 results could be explained due to the different number of participants in the sample, or due to the different pathology of the participants; in this study, only patients with AD were included, while in the study conducted by Shigihara et al. (2020b) the sample was composed of AD and vascular dementia patients.

### Limitations and Future Lines

Although this study has yielded interesting findings, there are also some methodological issues that have to be mentioned, as this is an exploratory study intended to be continued in the future.

Firstly, the sample size is limited due to the difficulty of carrying out this type of study, that requires a longitudinal follow-up. This issue impacts, in turn, the stability of the networks, probably due to the usage of bootstrapping that, with reduced sample sizes (*N* = 19 in our case), yields high variability between iterations (Efron and Tibshirani, 1993). In order to minimize the impact on the stability of the networks, they have been generated considering only the statistically significant connections (Supplementary Figures 1–4). Nevertheless, we are working on incorporating new participants into the database, which could also be interesting to design a classification model useful to predict the responsiveness of a patient to the NPT.

Another limitation is that we collapsed all the ROIs, considering only the spatial dimension of the data by means of the SSE. The results obtained with the SSE-related measures support future studies that would address the role of spatial patterns in detail. By analyzing its influence in each ROI separately, deep insights on the NPT outcomes could be obtained.

We have used only two tests in the cognitive assessments. The inclusion of additional cognitive tests would be useful to increase the robustness of the results by diminishing the impact of biases and measurement errors. Besides, it would be also interesting to disaggregate the MMSE results in its different domains to assess how the NPT differently influences diverse cognitive domains.

Furthermore, we have obtained interesting findings by analyzing the association between the NPT outcome and local activation (spectral and non-linear) neurophysiological parameters. By analyzing the relationship between the NPT outcome and connectivity or graph parameters in future studies, we could potentially obtain a broader characterization of the neurophysiological patterns associated with the NPT.

Finally, we have conducted the study using resting-state MEG recordings, where the background brain activity is measured. Resting-state is a widely used paradigm, but it would be of great interest to replicate the analysis performed in this study using brain signals during sleep, as NPTs are able to ameliorate the sleep disturbances provoked by AD (Berry et al., 2012; Horvath, 2018; Zucchella et al., 2018).

## Conclusions

In this study, we conducted an exploratory analysis about the associations between different local activation neurophysiological parameters (spectral and non-linear, as well their spatial counterparts) and the NPT outcome, quantified with MMSE and DBD-13 tests. Our findings suggest that the NPT modifies the association network structure, influencing the behavioral disturbances and suggesting its relationship with the neurophysiological patterns. Changes in cognition and behavior due to the NPT are related to the spectral changes in MEG activity, especially in the beta band. Furthermore, the NPT induces spatial-dependent patterns in MEG activity that are able to reflect the cognitive and behavioral changes due to the therapeutic intervention. Finally, we can conclude that the analyzed neurophysiological parameters are potential predictors of the NPT outcome; specifically, less severe neurophysiological alterations due to AD can be associated with a better prognosis of the NPT.

## Data Availability Statement

The datasets presented in this study can be found in online repositories. The names of the repository/repositories and accession number(s) can be found below: all data generated during this study are available from Rodriguez, Victor (2021), “NPT parameters,” Mendeley Data, V1, doi: 10.17632/99pmshmz7m.1 (http://dx.doi.org/10.17632/99pmshmz7m.1). The raw signals analyzed during the current study are available from the authors upon reasonable request. Furthermore, the methodology is based on widespread well-documented pipelines; however, the code used in the analyses is also available from the authors under reasonable request.

## Ethics Statement

The studies involving human participants were reviewed and approved by Ethics Committee of Hokuto Hospital, Hokuto Hospital, Obihiro, Japan. The patients/participants provided their written informed consent to participate in this study.

## Author Contributions

VR-G: managed the study, developed the software and the data visualizations, managed the data management, interpreted the results, and wrote the manuscript. CG: conceptualized the study, interpreted the results, reviewed and edited the manuscript. HH and YS: collected the data, interpreted the results, reviewed and edited the manuscript. RH: interpreted the results, reviewed and edited the manuscript. JP: managed the study, conceptualized the study, interpreted the results, reviewed and edited the manuscript. All authors contributed to the article and approved the submitted version.

## Conflict of Interest

HH was employed by the company RICOH Company, Ltd. The remaining authors declare that the research was conducted in the absence of any commercial or financial relationships that could be construed as a potential conflict of interest.

## Publisher’s Note

All claims expressed in this article are solely those of the authors and do not necessarily represent those of their affiliated organizations, or those of the publisher, the editors and the reviewers. Any product that may be evaluated in this article, or claim that may be made by its manufacturer, is not guaranteed or endorsed by the publisher.

## Footnotes

^1^ http://neuroimage.usc.edu/brainstorm

^2^ https://gephi.org/

## References

[B1] Alzheimer’s Association (2019). 2019 Alzheimer’s disease facts and figures. Alzheimer’s Dement. 15, 321–387. 10.1016/j.jalz.2019.01.010

[B2] Alzheimer’s Disease International (2019). World Alzheimer report 2019: Attitudes to Dementia. London: Alzheimer’s Disease International. Available online at: https://www.alzint.org/u/WorldAlzheimerReport2019.pdf.

[B3] Amjad I. Toor H. Niazi I. K. Afzal H. Jochumsen M. Shafique M.. (2019). Therapeutic effects of aerobic exercise on EEG parameters and higher cognitive functions in mild cognitive impairment patients. Int. J. Neurosci. 129, 551–562. 10.1080/00207454.2018.1551894 30929591

[B4] Anderson K. Grossberg G. T. (2014). Brain games to slow cognitive decline in Alzheimer’s disease. J. Am. Med. Dir. Assoc. 15, 536–537. 10.1016/j.jamda.2014.04.014 24913208

[B5] Augustine J. R. (2007). Human Neuroanatomy, 1st Edn. Amsterdam, Netherlands: Elsevier.

[B6] Başar E. Femir B. Emek-Savaş D. D. Güntekin B. Yener G. G. (2017). Increased long distance event-related gamma band connectivity in Alzheimer’s disease. Neuroimage Clin. 14, 580–590. 10.1016/j.nicl.2017.02.021 28367402 PMC5361871

[B7] Babiloni C. Ferri R. Moretti D. V. Strambi A. Binetti G. Dal Forno G.. (2004). Abnormal fronto-parietal coupling of brain rhythms in mild Alzheimer’s disease: a multicentric EEG study. Eur. J. Neurosci. 19, 2583–2590. 10.1111/j.0953-816X.2004.03333.x 15128412

[B8] Babiloni C. Pizzella V. Gratta C. D. Ferretti A. Romani G. L. (2009). Fundamentals of electroencefalography, magnetoencefalography and functional magnetic resonance imaging. Int. Rev. Neurobiol. 86, 67–80. 10.1016/S0074-7742(09)86005-4 19607991

[B9] Barberán A. Bates S. T. Casamayor E. O. Fierer N. (2012). Using network analysis to explore co-occurrence patterns in soil microbial communities. ISME J. 6, 343–351. 10.1038/ismej.2011.119 21900968 PMC3260507

[B10] Bartos M. Vida I. Jonas P. (2007). Synaptic mechanisms of synchronized gamma oscillations in inhibitory interneuron networks. Nat. Rev. Neurosci. 8, 45–56. 10.1038/nrn2044 17180162

[B11] Baumgarten M. Becker R. Gauthier S. (1990). Validity and reliability of the dementia behavior disturbance scale. J. Am. Geriatr. Soc. 38, 221–226. 10.1111/j.1532-5415.1990.tb03495.x 2313003

[B12] Berry R. B. Budhiraja R. Gottlieb D. J. Gozal D. Iber C. Kapur V. K.. (2012). Rules for scoring respiratory events in sleep: update of the 2007 AASM manual for the scoring of sleep and associated events. deliberations of the sleep apnea definitions task force of the american academy of sleep medicine. J. Clin. Sleep Med. 8, 597–619. 10.5664/jcsm.2172 23066376 PMC3459210

[B13] Blackman R. B. Tukey J. W. (1958). The measurement of power spectra from the point of view of communications engineering. Bell System Techn. J. 37, 185–282.

[B14] Borsboom D. (2017). A network theory of mental disorders. World Psychiatry 16, 5–13. 10.1002/wps.20375 28127906 PMC5269502

[B15] Borsboom D. Cramer A. O. J. (2013). Network analysis: an integrative approach to the structure of psychopathology. Ann. Rev. Clin. Psychol. 9, 91–121. 10.1146/annurev-clinpsy-050212-185608 23537483

[B16] Bruña R. Poza J. Gómez C. García M. Fernández A. Hornero R. (2012). Analysis of spontaneous MEG activity in mild cognitive impairment and Alzheimer’s disease using spectral entropies and statistical complexity measures. J. Neural Eng. 9:036007. 10.1088/1741-2560/9/3/036007 22571870

[B17] Cotelli M. Manenti R. Zanetti O. (2012). Reminiscence therapy in dementia: a review. Maturitas 72, 203–205. 10.1016/j.maturitas.2012.04.008 22607813

[B18] Cummings J. L. (2003). The Neuropsychiatry of Alzheimer’s Disease and Related Dementias, 1st Edn. Boca Raton, FL: CRC Press.

[B19] Dauwels J. Srinivasan K. Ramasubba Reddy M. Musha T. Vialatte F.-B. Latchoumane C.. (2011). Slowing and loss of complexity in Alzheimer’s EEG: two sides of the same coin. Int. J. Alzheimer’s Dis. 2011:539621. 10.4061/2011/539621 21584257 PMC3090755

[B20] Desikan R. S. Ségonne F. Fischl B. Quinn B. T. Dickerson B. C. Blacker D.. (2006). An automated labeling system for subdividing the human cerebral cortex on MRI scans into gyral based regions of interest. Neuroimage 31, 968–980. 10.1016/j.neuroimage.2006.01.021 16530430

[B21] Douw L. Nieboer D. Stam C. J. Tewarie P. Hillebrand A. (2018). Consistency of magnetoencephalographic functional connectivity and network reconstruction using a template versus native MRI for co-registration. Hum. Brain Mapp. 39, 104–119. 10.1002/hbm.23827 28990264 PMC5725722

[B22] Dyer S. M. Harrison S. L. Laver K. Whitehead C. Crotty M. (2018). An overview of systematic reviews of pharmacological and non-pharmacological interventions for the treatment of behavioral and psychological symptoms of dementia. Int. Psychogeriatr. 30, 295–309. 10.1017/S1041610217002344 29143695

[B23] Efron B. (1992). “Bootstrap methods: another look at the jackknife,” in Breakthroughs in Statistics, 1st edition, eds Kotz S. Johnson N. L. (Berlin, Germany: Springer), 569–593.

[B24] Efron B. Tibshirani R. J. (1993). An Introduction to the Bootstrap, 1st edition. Boca Raton, FL: Chapman and Hall/CRC.

[B25] Engels M. M. A. van der Flier W. M. Stam C. J. Hillebrand A. Scheltens P. van Straaten E. C. W. (2017). Alzheimer’s disease: the state of the art in resting-state magnetoencephalography. Clin. Neurophysiol. 128, 1426–1437. 10.1016/j.clinph.2017.05.012 28622527

[B26] Epskamp S. Borsboom D. Fried E. I. (2018). Estimating psychological networks and their accuracy: a tutorial paper. Behav. Res. Methods 50, 195–212. 10.3758/s13428-017-0862-1 28342071 PMC5809547

[B27] Escudero J. Hornero R. Abásolo D. Fernández A. (2009). Blind source separation to enhance spectral and non-linear features of magnetoencephalogram recordings. Application to Alzheimer’s disease. Med. Eng. Phys. 31, 872–879. 10.1016/j.medengphy.2009.04.003 19482539

[B28] Fernández A. Hornero R. Gómez C. Turrero A. Gil-Gregorio P. Matías-Santos J.. (2010). Complexity analysis of spontaneous brain activity in alzheimer disease and mild cognitive impairment. Alzheimer Dis. Assoc. Disord. 24, 182–189. 10.1097/WAD.0b013e3181c727f7 20505435

[B29] Fernández A. Hornero R. Mayo A. Poza J. Gil-Gregorio P. Ortiz T. (2006). MEG spectral profile in Alzheimer’s disease and mild cognitive impairment. Clin. Neurophysiol. 117, 306–314. 10.1016/j.clinph.2005.10.017 16386951

[B30] Fernández A. Ríos-Lago M. Abásolo D. Hornero R. Álvarez-Linera J. Paul N.. (2011). The correlation between white-matter microstructure and the complexity of spontaneous brain activity: a difussion tensor imaging-MEG study. Neuroimage 57, 1300–1307. 10.1016/j.neuroimage.2011.05.079 21683794

[B31] Fernández A. Turrero A. Zuluaga P. Gil-Gregorio P. del Pozo F. Maestu F.. (2013). MEG delta mapping along the healthy aging-Alzheimer’s disease continuum: diagnostic implications. J. Alzheimer’s Dis. 35, 495–507. 10.3233/JAD-121912 23478303

[B32] Folstein M. F. Folstein S. E. McHugh P. R. (1975). “Mini-mental state”: a practical method for grading the cognitive state of patients for the clinician. J. Psychiatric Res. 12, 189–198. 10.1016/0022-3956(75)90026-6 1202204

[B33] Fonov V. S. Evans A. C. McKinstry R. C. Almli C. R. Collins D. L. (2009). Unbiased nonlinear average age-appropriate brain templates from birth to adulthood. NeuroImage 47:S102. 10.1016/S1053-8119(09)70884-5

[B34] Fornito A. Zalesky A. Bullmore E. T. (2016). Fundamentals of Brain Network Analysis, 1st Edn. Amsterdam, Netherlands: Elsevier.

[B35] Gómez C. Hornero R. (2010). Entropy and complexity analyses in Alzheimer’s disease: an MEG study. Open Biomed. Eng. J. 4, 223–235. 10.2174/1874120701004010223 21625647 PMC3044892

[B36] Gómez C. Hornero R. Abásolo D. Fernández A. Escudero J. (2009). Analysis of MEG background activity in Alzheimer’s disease using nonlinear methods and ANFIS. Ann. Biomed. Eng. 37, 586–594. 10.1007/s10439-008-9633-6 19130227

[B37] Gómez C. Hornero R. Abásolo D. Fernández A. López M. (2006). Complexity analysis of the magnetoencephalogram background activity in Alzheimer’s disease patients. Med. Eng. Phys. 28, 851–859. 10.1016/j.medengphy.2006.01.003 16503184

[B38] Gramfort A. Papadopoulo T. Olivi E. Clerc M. (2010). OpenMEEG: opensource software for quasistatic bioelectromagnetics. BioMed. Eng. Online 9:45. 10.1186/1475-925X-9-45 20819204 PMC2949879

[B39] Griffen T. C. Maffei A. (2014). GABAergic synapses: their plasticity and role in sensory cortex. Front. Cell. Neurosci. 8:91. 10.3389/fncel.2014.00091 24723851 PMC3972456

[B40] Hornero R. Abásolo D. Escudero J. Gómez C. (2009). Nonlinear analysis of electroencephalogram and magnetoencephalogram recordings in patients with Alzheimer’s disease. Philos. Trans. A Math. Phys. Eng. Sci. 367, 317–336. 10.1098/rsta.2008.0197 18940776

[B41] Hornero R. Escudero J. Fernández A. Poza J. Gómez C. (2008). Spectral and nonlinear analyses of MEG background activity in patients with Alzheimer’s disease. IEEE Trans. Biomed. Eng. 55, 1658–1665. 10.1109/tbme.2008.919872 18714829

[B42] Horvath A. (2018). EEG and ERP biomarkers of Alzheimer’s disease a critical review. Front. Biosci. (Landmark Ed) 23, 183–220. 10.2741/4587 28930543

[B43] Hsu T.-J. Tsai H.-T. Hwang A.-C. Chen L.-Y. Chen L.-K. (2017). Predictors of non-pharmacological intervention effect on cognitive function and behavioral and psychological symptoms of older people with dementia. Geriatr. Gerontol. Int. 17, 28–35. 10.1111/ggi.13037 28436192

[B44] Jacomy M. Venturini T. Heymann S. Bastian M. (2014). ForceAtlas2, a Continuous graph layout algorithm for handy network visualization designed for the gephi software. PLoS One 9:e98679. 10.1371/journal.pone.0098679 24914678 PMC4051631

[B45] Jensen O. Goel P. Kopell N. Pohja M. Hari R. Ermentrout B. (2005). On the human sensorimotor-cortex beta rhythm: sources and modeling. Neuroimage 26, 347–355. 10.1016/j.neuroimage.2005.02.008 15907295

[B46] Jeong J. (2004). EEG dynamics in patients with Alzheimer’s disease. Clin. Neurophysiol. 115, 1490–1505. 10.1016/j.clinph.2004.01.001 15203050

[B47] Jimeno N. Gomez-Pilar J. Poza J. Hornero R. Vogeley K. Meisenzahl E.. (2020). Main symptomatic treatment targets in suspected and early psychosis: new insights from network analysis. Schizophr. Bull. 46, 884–895. 10.1093/schbul/sbz140 32010940 PMC7345824

[B48] Klimesch W. (1999). EEG alpha and theta oscillations reflect cognitive and memory performance: a review and analysis. Brain Res. Rev. 29, 169–195. 10.1016/s0165-0173(98)00056-3 10209231

[B49] Knyazeva M. G. Jalili M. Brioschi A. Bourquin I. Fornari E. Hasler M.. (2010). Topography of EEG multivariate phase synchronization in early Alzheimer’s disease. Neurobiol. Aging 31, 1132–1144. 10.1016/j.neurobiolaging.2008.07.019 18774201

[B50] Kurz A. F. Leucht S. Lautenschlager N. T. (2011). The clinical significance of cognition-focused interventions for cognitively impaired older adults: A systematic review of randomized controlled trials. Int. Psychogeriatr. 23, 1364–1375. 10.1017/S1041610211001001 21740614

[B51] Lai M. Demuru M. Hillebrand A. Fraschini M. (2018). A comparison between scalp- and source-reconstructed EEG networks. Sci. Rep. 8:12269. 10.1038/s41598-018-30869-w 30115955 PMC6095906

[B52] Lanctôt K. L. Herrmann N. Mazzotta P. Khan L. R. Ingber N. (2004). GABAergic function in Alzheimer’s disease: evidence for dysfunction and potential as a therapeutic target for the treatment of behavioural and psychological symptoms of dementia. Can. J. Psychiatry 49, 439–453. 10.1177/070674370404900705 15362248

[B53] Lempel A. Ziv J. (1976). On the complexity of finite sequences. IEEE Trans. Informat. Theory 22, 75–81.

[B54] Lin F.-H. Witzel T. Hämäläinen M. S. Dale A. M. Belliveau J. W. Stufflebeam S. M. (2004). Spectral spatiotemporal imaging of cortical oscillations and interactions in the human brain. Neuroimage 23, 582–595. 10.1016/j.neuroimage.2004.04.027 15488408 PMC2884198

[B55] Lu L.-C. Lan S.-H. Hsieh Y.-P. Yen Y.-Y. Chen J.-C. Lan S.-J. (2020). Horticultural therapy in patients with dementia: a systematic review and meta-analysis. Am. J. Alzheimers Dis. Other Demen. 35:1533317519883498. 10.1177/1533317519883498 31690084 PMC10623907

[B56] Mably A. J. Colgin L. L. (2018). Gamma oscillations in cognitive disorders. Curr. Opin. Neurobiol. 52, 182–187. 10.1016/j.conb.2018.07.009 30121451 PMC6139067

[B57] Machida A. (2012). Estimation of the reliability and validity of the short version of the 28-item dementia behavior disturbance scale. Nippon Ronen Igakkai Zasshi. Jpn. J. Geriatr. 49, 463–467. 10.3143/geriatrics.49.463 23269026

[B58] Mahjoory K. Nikulin V. V. Botrel L. Linkenkaer-Hansen K. Fato M. M. Haufe S. (2017). Consistency of EEG source localization and connectivity estimates. Neuroimage 152, 590–601. 10.1016/j.neuroimage.2017.02.076 28300640

[B59] Maki Y. Sakurai T. Okochi J. Yamaguchi H. Toba K. (2018). Rehabilitation to live better with dementia. Geriatr. Gerontol. Int. 18, 1529–1536. 10.1111/ggi.13517 30318671

[B60] Mandal P. K. Banerjee A. Tripathi M. Sharma A. (2018). A comprehensive review of magnetoencephalography (MEG) studies for brain functionality in healthy aging and Alzheimer’s disease (AD). Front. Computat. Neurosci. 12:60. 10.3389/fncom.2018.00060 30190674 PMC6115612

[B61] Martorell A. J. Paulson A. L. Suk H.-J. Abdurrob F. Drummond G. T. Guan W.. (2019). Multi-sensory gamma stimulation ameliorates Alzheimer’s-associated pathology and improves cognition. Cell 177, 256–271.e22. 10.1016/j.cell.2019.02.014 30879788 PMC6774262

[B62] McGrattan A. M. McGuinness B. McKinley M. C. Kee F. Passmore P. Woodside J. V.. (2019). Diet and inflammation in cognitive ageing and Alzheimer’s disease. Curr. Nutr. Rep. 8, 53–65. 10.1007/s13668-019-0271-4 30949921 PMC6486891

[B63] McKhann G. M. Knopman D. S. Chertkow H. Hyman B. T. Jack C. R. Kawas C. H.. (2011). The diagnosis of dementia due to Alzheimer’s disease: recommendations from the national institute on aging-Alzheimer’s association workgroups on diagnostic guidelines for Alzheimer’s disease. Alzheimer’s Dement. 7, 263–269. 10.1016/j.jalz.2011.03.005 21514250 PMC3312024

[B64] Mutanen T. P. Metsomaa J. Liljander S. Ilmoniemi R. J. (2018). Automatic and robust noise suppression in EEG and MEG: the SOUND algorithm. Neuroimage 166, 135–151. 10.1016/j.neuroimage.2017.10.021 29061529

[B65] Nagamatsu L. S. Handy T. C. Hsu C. L. Voss M. Liu-Ambrose T. (2012). Resistance training promotes cognitive and functional brain plasticity in seniors with probable mild cognitive impairment. Arch. Int. Med. 172, 666–668. 10.1001/archinternmed.2012.379 22529236 PMC3514552

[B66] Nakamura A. Cuesta P. Fernández A. Arahata Y. Iwata K. Kuratsubo I.. (2018). Electromagnetic signatures of the preclinical and prodromal stages of Alzheimer’s disease. Brain 141, 1470–1485. 10.1093/brain/awy044 29522156 PMC5920328

[B67] Oliveira A. M. D. Radanovic M. Mello P. C. H. D. Buchain P. C. Vizzotto A. D. B. Celestino D. L.. (2015). Nonpharmacological interventions to reduce behavioral and psychological symptoms of dementia: a systematic review. Biomed. Res. Int. 2015:218980. 10.1155/2015/218980 26693477 PMC4676992

[B68] Poil S.-S. de Haan W. van der Flier W. M. Mansvelder H. D. Scheltens P. Linkenkaer-Hansen K. (2013). Integrative EEG biomarkers predict progression to Alzheimer’s disease at the MCI stage. Front. Aging Neurosci. 5:58. 10.3389/fnagi.2013.00058 24106478 PMC3789214

[B69] Porges E. C. Woods A. J. Edden R. A. E. Puts N. A. J. Harris A. D. Chen H.. (2017). Frontal gamma-aminobutyric acid concentrations are associated with cognitive performance in older adults. Biol. Psychiatry Cogn. Neurosci. Neuroimaging 2, 38–44. 10.1016/j.bpsc.2016.06.004 28217759 PMC5312683

[B70] Poza J. Gómez C. Bachiller A. Hornero R. (2012). Spectral and non-linear analyses of spontaneous magnetoencephalographic activity in Alzheimer’s disease. J. Healthcare Eng. 3, 299–322. 10.1260/2040-2295.3.2.299

[B71] Poza J. Hornero R. Abásolo D. Fernández A. García M. (2007). Extraction of spectral based measures from MEG background oscillations in Alzheimer’s disease. Med. Eng. Phys. 29, 1073–1083. 10.1016/j.medengphy.2006.11.006 17204443

[B72] Poza J. Hornero R. Abásolo D. Fernández A. Mayo A. (2008a). Evaluation of spectral ratio measures from spontaneous MEG recordings in patients with Alzheimer’s disease. Comput. Methods Programs Biomed. 90, 137–147. 10.1016/j.cmpb.2007.12.004 18249462

[B73] Poza J. Hornero R. Escudero J. Fernández A. Sánchez C. I. (2008b). Regional analysis of spontaneous MEG rhythms in patients with Alzheimer’s disease using spectral entropies. Ann. Biomed. Eng. 36, 141–152. 10.1007/s10439-007-9402-y 17994279

[B74] Qaseem A. Snow V. Cross T. Forciea M. A. Hopkins R. Shekelle P.. (2008). Current pharmacologic treatment of dementia: a clinical practice guideline from the american college of physicians and the american academy of family physicians. Ann. Int. Med. 148, 370–378. 10.7326/0003-4819-148-5-200803040-00008 18316755

[B75] Raji C. A. Lopez O. L. Kuller L. H. Carmichael O. T. Becker J. T. (2009). Age, Alzheimer disease and brain structure. Neurology 73, 1899–1905. 10.1212/WNL.0b013e3181c3f293 19846828 PMC2788799

[B76] Rege S. Geetha T. Broderick T. Babu J. (2016). Can diet and physical activity limit Alzheimer’s disease risk. Curr. Alzheimer Res. 14, 76–93. 10.2174/1567205013666160314145700 26971938

[B77] Rodríguez-González V. Gómez C. Shigihara Y. Hoshi H. Revilla-Vallejo M. Hornero R.. (2020). Consistency of local activation parameters at sensor- and source-level in neural signals. J. Neural Eng. 17:056020. 10.1088/1741-2552/abb582 33055364

[B78] Rodríguez-González V. Poza J. Núñez P. Gómez C. García M. Shigihara Y.. (2019). Towards automatic artifact rejection in resting-state MEG recordings: evaluating the performance of the SOUND algorithm. Annu. Int. Conf. IEEE Eng. Med. Biol. Soc. 2019, 4807–4810. 10.1109/EMBC.2019.8856587 31946937

[B79] Roh J. H. Park M. H. Ko D. Park K.-W. Lee D.-H. Han C.. (2011). Region and frequency specific changes of spectral power in Alzheimer’s disease and mild cognitive impairment. Clin. Neurophysiol. 122, 2169–2176. 10.1016/j.clinph.2011.03.023 21715226

[B80] Ruiz-Gómez S. Gómez C. Poza J. Gutiérrez-Tobal G. Tola-Arribas M. Cano M.. (2018). Automated multiclass classification of spontaneous EEG activity in Alzheimer’s disease and mild cognitive impairment. Entropy(Basel) 20:35. 10.3390/e20010035 33265122 PMC7512207

[B81] Shigihara Y. Hoshi H. Poza J. Rodríguez-González V. Gómez C. Kanzawa T. (2020a). Predicting the outcome of non-pharmacological treatment for patients with dementia-related mild cognitive impairment. Aging (Albany NY) 12, 24101–24116. 10.18632/aging.202270 33289701 PMC7762505

[B82] Shigihara Y. Hoshi H. Shinada K. Okada T. Kamada H. (2020b). Non-pharmacological treatment changes brain activity in patients with dementia. Sci. Rep. 10:6744. 10.1038/s41598-020-63881-0 32317774 PMC7174400

[B83] Stam C. J. (2005). Nonlinear dynamical analysis of EEG and MEG: review of an emerging field. Clin. Neurophysiol. 116, 2266–2301. 10.1016/j.clinph.2005.06.011 16115797

[B84] Stam C. J. van Straaten E. C. W. (2012). The organization of physiological brain networks. Clin. Neurophysiol. 123, 1067–1087. 10.1016/j.clinph.2012.01.011 22356937

[B85] Sugishita M. Hemmi I. Iwatsubo T. (2010). Japanese versions equivalent to original english neuropsychological tests in ADNI. Alzheimer’s Dement. 6, S348–S348. 10.1016/j.jalz.2010.05.1166

[B86] Tadel F. Baillet S. Mosher J. C. Pantazis D. Leahy R. M. (2011). Brainstorm: a user-friendly application for MEG/EEG analysis. Comput. Intell. Neurosci. 2011:879716. 10.1155/2011/879716 21584256 PMC3090754

[B87] Uhlhaas P. J. Haenschel C. Nikolic D. Singer W. (2008). The role of oscillations and synchrony in cortical networks and their putative relevance for the pathophysiology of schizophrenia. Schizophr. Bull. 34, 927–943. 10.1093/schbul/sbn062 18562344 PMC2632472

[B88] van Deursen J. A. Vuurman E. F. P. M. Verhey F. R. J. van Kranen-Mastenbroek V. H. J. M. Riedel W. J. (2008). Increased EEG gamma band activity in Alzheimer’s disease and mild cognitive impairment. J. Neural Transm. (Vienna) 115, 1301–1311. 10.1007/s00702-008-0083-y 18607528 PMC2525849

[B89] Wang J. Fang Y. Wang X. Yang H. Yu X. Wang H. (2017). Enhanced gamma activity and cross-frequency interaction of resting-state electroencephalographic oscillations in patients with Alzheimer’s disease. Front. Aging Neurosci. 9:243. 10.3389/fnagi.2017.00243 28798683 PMC5526997

[B90] Zhang W. J. (2011). Constructing ecological interaction networks by correlation analysis: hints from community sampling. Network Biol. 1, 81–98. 10.0000/issn-2220-8879-networkbiology-2011-v1-0008

[B91] Zucchella C. Sinforiani E. Tamburin S. Federico A. Mantovani E. Bernini S.. (2018). The multidisciplinary approach to Alzheimer’s disease and dementia. a narrative review of non-pharmacological treatment. Front. Neurol. 9:1058. 10.3389/fneur.2018.01058 30619031 PMC6300511

