# Supplementary material for: Exploring the Interactions Between Neurophysiology and Cognitive and Behavioral Changes Induced by a Non-pharmacological Treatment: A Network Approach
Source: Front Aging Neurosci. 2021 Jul 29;13:696174. doi: 10.3389/fnagi.2021.696174 (PMC8358307; doi:10.3389/fnagi.2021.696174)
Supplement: Supplementary file 1 [file Data_Sheet_1.PDF]

Supplementary Material

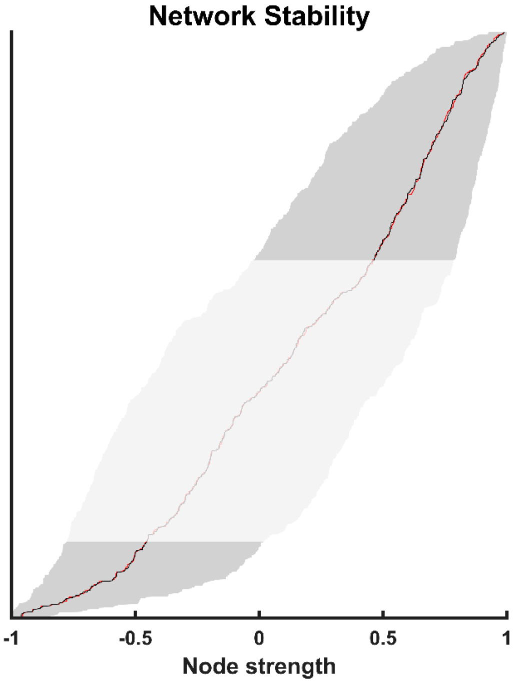

Figure S1. Edge stability of the Pre-NPT network. The horizontal axis depicts the weight of the edge between each pair of nodes, that are represented on the vertical axis top-down ordered. Grey shadow indicates the bootstrapped confidence interval (95%) of the edge weights, whereas black and red lines correspond to the mean of the bootstrapped sample and the real value of the Pre-NPT network, respectively. The shaded region delimitates the non-significant connections that were not considered to generate the association network. This interval was estimated by removing the non-significant values from the bootstrapped sample and obtaining the median values, thereby where a gap between ρ=-0.458 and ρ=0.465 values can be observed.

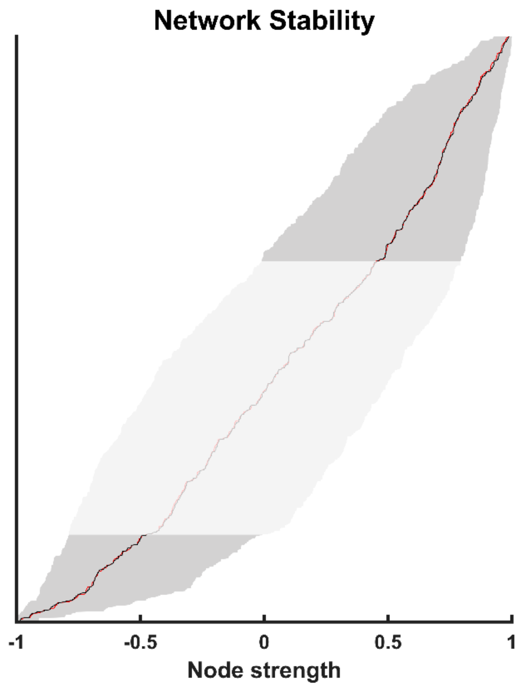

Figure S2. Edge stability of the Post-NPT network. The horizontal axis depicts the weight of the edge between each pair of nodes, that are represented on the vertical axis top-down ordered. Grey shadow indicates the bootstrapped confidence interval (95%) of the edge weights, whereas black and red lines correspond to the mean of the bootstrapped sample and the real value of the Post-NPT network, respectively. The shaded region delimitates the non-significant connections that were not considered to generate the association network This interval was estimated by removing the non-significant values from the bootstrapped sample and obtaining the median values, thereby where a gap between ρ=-0.457 and ρ=0.457 values can be observed.

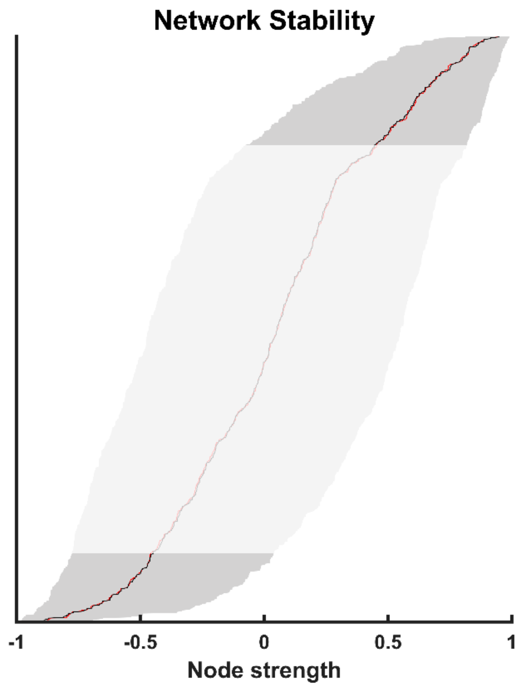

Figure S3. Edge stability of the network of changes. The horizontal axis depicts the weight of the edge between each pair of nodes, that are represented on the vertical axis top-down ordered. Grey shadow indicates the bootstrapped confidence interval (95%) of the edge weights, whereas black and red lines correspond to the mean of the bootstrapped sample and the real value of the network of changes, respectively. The shaded region delimitates the non-significant connections that were not considered to generate the association network. This interval was estimated by removing the non-significant values from the bootstrapped sample and obtaining the median values, thereby where a gap between ρ=-0.457 and ρ=0.456 values can be observed.

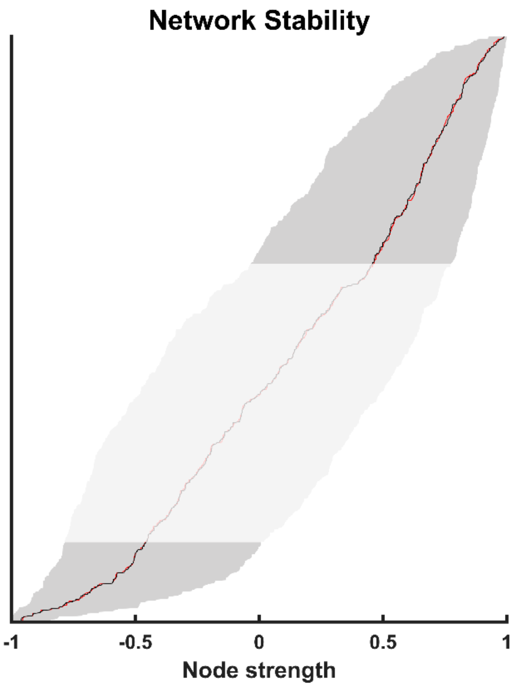

Figure S4. Edge stability of the Prediction network. The horizontal axis depicts the weight of the edge between each pair of nodes, that are represented on the vertical axis top-down ordered. Grey shadow indicates the bootstrapped confidence interval (95%) of the edge weights, whereas black and red lines correspond to the mean of the bootstrapped sample and the real value of the Prediction network, respectively. The shaded region delimitates the non-significant connections that were not considered to generate the association network. This interval was estimated by removing the non-significant values from the bootstrapped sample and obtaining the median values, thereby where a gap between ρ=-0.461 and ρ=0.460 values can be observed.
